# Cell Surface-Associated Proteins in the Filamentous Cyanobacterium *Anabaena* sp. strain PCC 7120

**DOI:** 10.1264/jsme2.ME12091

**Published:** 2012-10-10

**Authors:** Hidehisa Yoshimura, Masahiko Ikeuchi, Masayuki Ohomori

**Affiliations:** 1 Department of Life Sciences, Graduate School of Arts and Sciences, The University of Tokyo, 3–8–1 Komaba, Meguro-ku, Tokyo 153–8902, Japan; 2 Department of Biological Science, Faculty of Science and Engineering, Chuo University, 1–13–27 Kasuga, Bunkyo-ku, Tokyo 112–8551, Japan

**Keywords:** cyanobacteria, cell surface protein, environmental response, stress response

## Abstract

The cell surface senses environmental changes first and transfers signals into the cell. To understand the response to environmental changes, it is necessary to analyze cell surface components, particularly cell surface-associated proteins. We therefore investigated cell surface-associated proteins from the filamentous cyanobacterium *Anabaena* sp. strain PCC 7120. The cell surface-associated proteins extracted by an acidic buffer were resolved by SDS-PAGE. Eighteen proteins were identified from resolved bands by amino-terminal sequencing. Analysis of cell surface-associated proteins indicated that several proteins among them were involved in nucleic acid binding, protein synthesis, proteolytic activity and electron transfer, and other proteins were involved in the stress response.

Cyanobacteria are Gram-negative bacteria possessing the unifying property of performing oxygenic photosynthesis and the ancestor of plant chloroplasts. Microorganisms, including cyanobacteria, are faced with extreme environments and the cell surface structure serves as a dynamic interface between microorganisms and the external environment, and as a protection against environmental stresses (31). The cell surface components are composed of polysaccharides, cell surface-associated proteins, pigments, *etc* (31, 32). Cyanobacteria produce polysaccharides and secrete to the cell surface. Extracellular polysaccharides play protective roles in some stresses (38, 47, 49). Extracellular pigments are linked to the cell surface and have been reported to serve as a screen for protection against UV stress (2); therefore, determination of the cell surface components is very important to elucidate the whole mechanism of the environmental response. Proteomic analysis of protein extract from isolated cytoplasmic membrane, periplasm, and the outer membrane has recently been accomplished in cyanobacteria (6, 13, 14, 23); however, little is known about cyanobacterial cell surface-associated proteins outside of the outer membrane. In the present work, we extracted cell surface-associated proteins of the filamentous *Anabaena* sp. strain PCC 7120 (also called, *Nostoc* sp. strain PCC 7120) and analyzed the major proteins among them.

*Anabaena* sp. strain PCC 7120 was grown in BG11 liquid culture medium (35) with 20 mM *N*-2-hydroxyethylpiperazine-*N*′-2-ethanesulfonic acid (HEPES)-NaOH (pH 7.5) at 30°C under continuous illumination provided by fluorescent lamps at 30 μmol photons m^−2^s^−1^. Liquid cultures were bubbled with air containing 1% (v/v) CO_2_.

Cell surface-associated proteins in Gram-negative bacteria are not anchored to the cell surface via a covalent interaction but interact with a component of the outer membrane, such as lipopolysaccharides, via a cation-mediated interaction (32). Cell surface-associated proteins are extractable from the cell envelope by several procedures, including treatments with detergents, urea, chelating agents, and acids (19). We failed to extract cell surface-associated proteins with various concentrations of SDS, urea and EDTA except acids. These procedures caused more or less cell rupture of *Anabaena* sp. strain PCC 7120 (data not shown). A low-pH solution that can break ionic bonds between cell surface-associated proteins and the component of the outer membrane is effective in extracting cell surface-associated proteins from the whole cells (44).

Cells (OD750 3.0–4.0) from liquid culture were harvested by centrifugation at 17,700×*g* for 10 min at room temperature. After harvest, the cells were washed once with BG11 liquid medium by centrifugation at 17,700×*g* for 3 min at room temperature. We incubated the harvested cells in 30 mM HEPES, pH 2.5–5.5 for 2 h at 37°C; however, the cell surface-associated protein was hardly extracted at pH 3.5 and contamination of phycocyanin or allophycocyanin by cell rupture was present in the extracts at pH 4.5 and pH 5.5. Therefore, the cells were incubated in 30 mM HEPES, pH 2.5, for 2 h at 37°C (44). No phycocyanin or allophycocyanin was detected in bands from 15–25 kDa by amino-terminal sequencing after SDS-PAGE in this condition. In addition, we compared with the SDS-PAGE profile of the extract from the same acidic condition using broken cells. The SDS-PAGE profile was highly similar to that of the whole cell extract (Fig. 1B) but was not similar to that of the extract of cell surface-associated proteins (Fig. 1A). After incubation, the cells were then removed by centrifugation at 3,000×*g* for 10 min at room temperature. This removal step was repeated three times. The supernatant was adjusted to pH 7.5 with NaOH and insoluble materials were removed by centrifugation at 17,700×*g* for 10 min. The final supernatant was lyophilized. After the lyophilized sample was dissolved in distilled water, the solution was dialyzed in 50 mM HEPES (pH 7.5) with a cellulose tube (1 kDa cutoff). The dialyzed solution was suspended in a SDS loading buffer (50 mM Tris-HCl pH 6.8, 2% (w/v) SDS, 10% (w/v) glycerol, 1% (v/v) β-mercaptoethanol, 12.5 mM EDTA, 0.02% (w/v) bromophenol blue) for SDS-PAGE. No difference was observed among the SDS-PAGE profiles of cell surface-associated proteins extracted from cells at OD750 0.5–4.0. Whole cell extract was prepared by boiling the mixture of cells with the SDS loading buffer for 5 minutes. These samples were resolved by 15% SDS-PAGE. Proteins were stained with Coomassie Brilliant Blue R-250.

Proteins in the gel after SDS-PAGE were transferred to a poly vinylidene difluoride (PVDF) membrane (GE Healthcare Bioscience, Tokyo, Japan). Proteins on the membrane were stained with amide black. Amino-terminal sequencing of the stained bands was performed with PPSQ-20 (Shimadzu, Kyoto, Japan).

Cell surface-associated proteins and whole cell extract from *Anabaena* sp. strain PCC 7120 were resolved by 1D SDS-PAGE. Twenty-one major bands were detected in the profile of the cell surface-associated proteins (Fig. 1A). The molecular mass of the major cell surface-associated proteins from *Anabaena* sp. strain PCC 7120 was less than 40 kDa (Fig. 1), although proteolysis of a some of the cell surface-associated proteins might occur during extraction. On the other hand, the majority of cell surface-associated proteins had a molecular mass of more than 60 kDa in the unicellular cyanobacterium *Synechocystis* sp. strain PCC 6803 (48). The profile of cell surface-associated protein is different between *Synechocystis* sp. strain PCC 6803 and *Anabaena* sp. strain PCC 7120. S-layer protein is well known as a cell surface-associated protein with a molecular mass of 40–200 kDa (33). This suggests that S-layer protein is present in *Synechocystis* sp. strain PCC 6803 but is not major in the cell surface-associated proteins of *Anabaena* sp. PCC 7120. It is generally accepted that S-layer proteins have many roles, *e.g.*, as protective coats, structures involved in cell adhesion and surface recognition, molecular sieves, molecular and ion traps, scaffolding for enzymes and virulence factors (32). On the other hand, *Anabaena* sp. strain PCC 7120 cells product a large amount of extracellular polysaccharides compared with that of *Synechocystis* sp. strain PCC 6803 cells (our unpublished data). Extracellular polysaccharides of *Nostoc* species play protective roles in harmful environments (38, 47, 49), and extracellular polysaccharides may function as a substitute for S-layer proteins. It is also conceivable that some proteins in cell surface-associated proteins are assembled into macromolecule structures on the cell surface as S-layer proteins and the structures might play important roles physiologically.

Eighteen proteins were identified from bands in SDS-PAGE by amino-terminal sequencing (Table 1). The observed apparent molecular mass of the cell surface-associated proteins well agreed with their theoretical molecular mass (Table 1). The apparent molecular mass of All4782-, Alr0806- and All3826-derived proteins (25.3, 27.3 and 35.6 kDa, respectively) was larger than the theoretical molecular mass (13.0, 14.9 and 22.4 kDa, respectively), suggesting that these proteins are modified with compounds such as lipids and sugars or could form tight dimers. In addition, their theoretical isoelectric points (p*I*) were either acidic (p*I* <6.0) or basic (p*I* >9.0). This result agrees with the results reported in microorganisms that most proteins secreted to the cell surface and into the extracellular medium were acidic or basic proteins (9, 15). We could not identify proteins in 6 bands. Band 3 contained multiple proteins. Protein(s) in band 11 was not transferred to a PVDF membrane. N-termini of proteins in band 9, 14, 19 and 20 were blocked.

Half of the identified 18 proteins showed the predicted signal peptide for secretion (Table 2). The predicted signal peptide of Alr0198-derived protein was as long as 97 residues (Table 2). The signal sequence contained a possible twin-arginine motif which is involved in the Tat pathway (25). Typical Tat-type signal peptide contains positively charged amino acids Lys or Arg and the twin-arginine motif R/K-RX-Φ-Φ (Φ is a hydrophobic residue) in the N-terminal region, a hydrophobic H-region following the N-terminal region, and a proline at position -6 and A-X-A sequence at positions −3 to −1 relative to the cleavage site (39); however, the predicted signal peptide of Alr0198-derived protein was considerably different from the typical Tat-type signal peptide. A possible twin-arginine motif was present in the C-terminal region of the whole signal peptide. Furthermore, an H-region following the twin-arginine motif was absent in the signal peptide. The long N-terminal region located before the possible twin-arginine motif may possess unknown motifs.

The typical Sec-type signal peptide that is involved in the Sec pathway (39) is lacking only the twin-arginine motif compared with Tat-type signal peptide. Alr0896-, All0459-, All1861- and Alr0114-derived proteins could be secreted by the Sec pathway.

The predicted signal peptide of Alr0600-, Alr0806-, Alr3276- and All3826-derived proteins did not contain the typical A-X-A sequence just before the cleavage site (Table 2). The signal peptide of Alr0806 was cleaved at the two positions between Met-23 and Thr-24 or between Thr-24 and Asn-25. Asr1134-, Asr4653-, Asr4319-, Asr3935-, Alr1718-, All0615-, All4201-, All4377- and All4782-derived proteins without signal peptides could be secreted by nonspecific and/or currently unknown translocation pathways (1).

Earlier studies have shown that both Sec and Tat pathways serve to export proteins across the plasma membrane in cyanobacteria (14, 23). Several ORFs corresponding to the subunits of Sec and Tat pathways are annotated in CyanoBase. The former ORFs are alr4851 to *secA*, all0121 to *secD*, asr5298 to *secE*, all0120 to *secF*, asl4181 to *secG* and all4197 to *secY*. The latter ORFs are asr3878 to *tatA/E*, asl0845 to *tatB*, all2456 to *tatC*, and alr1593 and all0420 to *tatD*. Since Sec and Tat translocons are located in the plasma membrane, other translocon(s) involved in the two-step protein secretion systems that are widespread in Gram-negative bacteria (5) would be required for translocation of proteins to the cell surface across the outer membrane.

Half of the 18 major proteins identified from the fraction of cell surface-associated proteins were strongly related to the stress response (Table 3). The gene expressions of *asr1134*, *asr4653*, *alr0198*, *alr0600*, *alr0896*, *all0459*, *alr1718*, *all4782* and *alr0806* are upregulated under drought stress (12). The gene expression of *all0459*, as part of the gene cluster from *all0457* to *all0459*, is also inducible under low temperature (28).

The protein sequence of Asr1134 and Asr4653 was similar to CsbD, which is widely distributed in bacteria (Table 3). CsbD is a bacterial general stress response protein and its expression is inducible under salt stress and phosphate-starvation stress in *Bacillus subtilis* (26). The protein sequence of Alr0198, All0459, All4782 and Alr0806 did not possess a hitherto-known domain at all and that of Alr0600 and Alr0896 showed very low similarity to a previously domain (Table 3). The protein sequence of Alr1718 was similar to YciG in other bacteria and contained a characteristic sequence motif K-G-G. YciG is expressed as part of an operon *yciGFE* (27), which is important in resistance to acid and salt stresses (42, 45). The protein sequence of All0615 was similar to YciF.

ORF alr0114 encodes a homologue of a plant chloroplast protein Tic22 that belongs to Tic complex, a translocator of the inner membrane (Table 3). Tic22 homologue (Slr0924) of *Synechocystis* sp. strain PCC 6803 has been found in the culture medium, periplasm and thylakoid (6, 7, 29). Furthermore, the expression levels in transcription and translation increased under salt and osmotic stresses (6, 30). These results suggest that Tic22 homologues play very important physiological roles at multiple locations. Other ORFs corresponding to subunits of Tic complex in *Arabidopsis thaliana* were present in the genome of *Anabaena* sp. strain PCC 7120. They are Alr5007 to Tic55 (E value of 2e-64), Alr1722 toTic32 (E value of 2e-31), All4804 to Tic20 (E value of 1e-08) and All4113 and All3977 to Tic21 (E values of 9e-15 and 2e-13, respectively). These homologues were not found in the outer membrane fraction (23) and our fraction of cell surface-associated proteins. The expression of *tic55* (*slr1747*) and *tic20* (*sll1737*) homologues of *Synechocystis* sp. strain PCC 6803 is responsible for UV-B stress, H_2_O_2_ treatment, cold stress and inorganic carbon limitation (13, 20, 24, 43). Cyanobacterial Tic homologues seem to be involved in not the translocation of proteins but also the stress response.

Unexpectedly, our fraction of cell surface-associated proteins contained proteins such as a histone-like DNA binding protein, HU, a subunit of 50S ribosomal protein, Rpl6, and a subunit of photosystem I, PsaE, that are commonly present intracellularly (Table 3). In order to check for contamination by cell lysis, we carried out Western blotting analysis with anti-D1 protein antibody (Supplementary Fig. S1). D1 protein is mainly present in thylakoids such as PsaE and Tic22. D1 protein was clearly detected in the whole cell extract. On the other hand, no D1 protein was detected in the fraction of cell surface-associated proteins. This result suggests that there was no contamination by cell lysis in the fraction.

ORF asr3935 encodes a histone-like DNA binding protein HU (Table 3). Histone-like proteins in bacteria are known to wrap DNA and restrain negative supercoiling and the resulting alterations affect several cellular processes (3). On the other hand, HlpA, a homologue of HU, of Gram-positive bacteria *Streptococcus* species localizes at the cell surface, interacting with extracellular polysaccharides (36). Mycobacterial histone-like DNA-binding proteins also localize at the cell surface and are involved in cell wall assembly (16, 34). A disruptant in *asr3935* (*hu*) exhibits slow growth, cellular fragility and the inability to differentiate heterocysts in *Anabaena* sp. strain PCC 7120 (18). The HU at the cell surface is probably involved in the assembly of the cell surface structure.

ORF all4377 encodes RNA binding protein RbpG (Table 3). RbpG contains a single RNA binding motif in the N-terminal region and a long C-terminal region that is absent from other paralogues and other organisms (11). The gene expression of *rbpG* is constitutive, while that of other paralogues is inducible by cold stress, and the mutant was incompletely segregated (4). RbpG is required for cell viability at the cell surface.

ORF all4201 encodes 50S ribosomal protein L6 Rpl6 (Table 3). Some ribosomal proteins have already been identified on the cell surface of *Saccharomyces cerevisiae* (15) and other microorganisms (22, 40). Ribosomal proteins are necessary for ribosome assembly and stability and it has been reported that some are involved in sensing environmental changes (41).

PsaE, a subunit of photosystem I complex, was found in the fraction of cell surface-associated proteins (Table 3). PsaE is commonly located on the cytosolic side of photosystem I in the thylakoid membrane and is involved in the stabilization of photosystem I, the anchoring of ferredoxin or flavodoxin, crosslinking with ferredoxin-NADP^+^ reductase and cyclic electron transport in cyanobacteria (8). It was recently revealed that electron transfer at the cell surface occurs between microorganisms and extracellular substrates such as iron and manganese oxides (10). PsaE might be involved in the anchoring and crosslinking of cell surface-associated proteins and contribute to the reduction of extracellular substrates at the cell surface.

ORF alr3276 encodes a protease composed of a functionally unknown region at the N-terminus and an M23 peptidase domain at the C-terminus (Table 3). Members of M23 peptidase family are zinc metallopeptidases and Gly-Gly endopeptidases. The M23 peptidase domain of CwlP in *Bacillus subtilis* exhibits peptidoglycan hydrolase activity (37). LasA of *Pseudomonas aeruginosa* is a protease possessing an M23 peptidase domain and is secreted into its environment as a virulence factor (17).

All1861- and All3826-derived proteins were also found in the outer membrane fraction of *Anabaena* sp. strain PCC 7120 (23) and contain two peptidoglycan binding-like domains. All1861-derived protein also contains a bacterial SH3-like domain. Bacterial SH3 domain is believed to promote survival in the invaded cell by modulating the pathways controlled by SH3 domains or invasion by binding to receptors on target cells (46). It has also been reported that the SH3 domain of the Gram-positive bacterium *Staphylococcus aureus* ALE-1 binds to the bacterial cell surface (21).

In summary, we analyzed non-covalently cell surface-associated proteins of the filamentous cyanobacterium *Anabaena* sp. strain PCC 7120. It was found that stress response-related but functionally unknown proteins were present at the cell surface. Furthermore, our findings suggested that some cytoplasmic proteins play important physiological roles at the cell surface. It has also been reported that many cytoplasmic proteins were associated with the cell surface of microorganisms, as mentioned above. Some cytoplasmic proteins detected in the fraction of cell surface-associated proteins are conceivably translocated from the cytoplasm to the cell surface depending on physiological and/or environmental conditions.

These findings suggest that an elaborate system on the cell surface is responsible for various environmental stresses and is involved in extracellular processes. More detailed cell surface proteome analysis and further studies to know the function of each cell surface-associated protein are necessary to elucidate the physiological role of cell surface-associated protein.

## Supplementary Material



## Figures and Tables

**Fig. 1:**
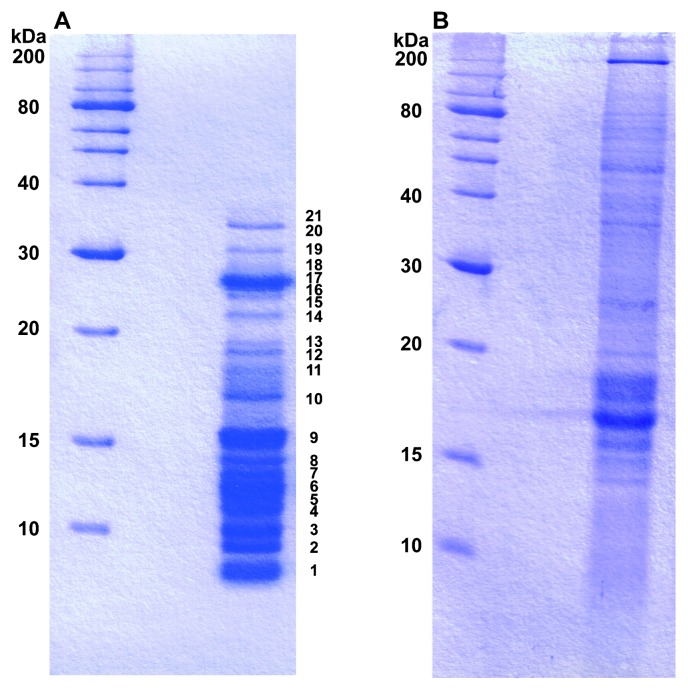
Profiles of cell surface-associated proteins (A) and whole cell extract (B) of *Anabaena* sp. strain PCC 7120. Each sample was loaded onto a 15% SDS-polyacrylamide gel for electrophoresis. Proteins in the gel were stained with Coomassie Brilliant Blue R-250. Bands are indicated by numbers and listed in Tables 1–3.

**Table 1:** Identified proteins in the cell surface fraction of *Anabaena* sp. strain PCC 7120^a^

band number	observed N-terminal sequence	predicted ORF	apparent molecular mass (kDa)	theoretical molecular mass (kDa)	theoretical p*I*
1	SXEDRAKATGKNIEG	asr1134	8.1	6.3	5.4
	SXEXRXEATAKNIEG	asr4653	8.1	6.4	5.4
2	VQRGSKVRILRPESY	asr4319	9.1	7.8	9.4
4	MXKGELVDAVAEKA	asr3935	10.6	10.1	9.6
5	ANREXELXYPGAEXP	alr0198	11.2	10.2	4.9
6	TSDATDSLXNXSXEF	alr0600	11.6	10.0	5.4
7	NTVQTPEGTYYKGT	alr0896	12.3	10.7	9.1
8	ISSYQSEPTEATDQL	all0459	13.0	9.0	4.7
10	SDTSKRGFAXMDE	alr1718	16.7	13.1	5.8
12	VQLSERPGTARINNF	all0615	20.2	20.6	5.7
	SRIGKRPITVPAKVQ	all4201	20.2	19.8	10.4
13	SVRLYIGNLPKEEID	all4377	20.9	18.2	9.2
15	AYVRTNGSXLNVRTS	all1861	25.3	21.4	11.4
	SEEIKPNSMEA	all4782	25.3	13.0	4.3
16	LSEQQIKEDLDSV	alr0114	26.3	26.9	9.6
17	T-NEPVKRATDSSEXA	alr0806	27.3	14.9	4.3
18	LQVQINPRNPRL	alr3276	28.4	28.4	10.2
21	DRQRNYTPQEFRTVL	all3826	35.6	22.4	9.8

a Molecular mass and p*I* were calculated using Expasy server http://www.expasy.ch/MW/pI calculator.

**Table 2:** Predicted signal peptides of cell surface-associated proteins in *Anabaena* sp. strain PCC 7120^a^

band number	ORF	predicted signal peptide^b^	length^c^
5	alr0198	MNSLMNVWE**R**L**R**L**R**QILTVFLAGILLIISTACSQGNPQGANPQNPAVQAGG ANNPYKNGGDKYVNSRFSTDPNITNPETKK**RR**DQANL**P**ISSQLLIA	97
6	alr0600	MADELD**K**V**K**IECFILS**K**LHSM**K**LNFSM**KK**LSNLG**R**WIATTVFCLSAIAFVW QGAFFADTSAMADNSTYLIA	71
7	alr0896	M**R**IFMN**R**VISWIQNILLRQILVVFLVAATFFVGQSFTYGTAMMAQA	46
8	all0459	M**K**IFSVALSML**R**PV**R**FLIVAFTCALLFLSSTV**P**AFA	36
15	all1861	MEFIAYSSMVIANQEANGQTEYLEYELP**K**FDFSWG**K**LL**K**SSA WLSVAGLMVLFTALTQVNGALA	64
16	alr0114	M**K**ALV**R**WGATLGLVGSTLLGTLSLGSL**P**AIA	31
17	alr0806	MIF**KKH**NLVLEVLTDFLTRTDFM/T	23/24
18	alr3276	MIT**K**IPNSNYQ**K**N**K**CSFDI**R**GE**KH**NYFLPVNLFIGFCAALPITLAL**P**VEA	50
21	all3826	MWCGFG**K**SSAVIATACVISASFVISNTTFA	31

a Signal peptides were predicted with our sequence results (Table 1) and the information based on CyanoBase (http://genome.kazusa.or.jp/cyanobase/Anabaena) and SignalP 4.0 (http://www.cbs.dtu.dk/services/SignalP/).

b Positively charged amino acids in the N-terminal region, twin-arginine and a proline residue around position -6 from the cleavage site in bold; H-region and the consensus sequence A-X-A in the C-terminal region are underlined.

c length, length of predicted signal peptide.

**Table 3:** List of family, domain or motif classified by Pfam (http://pfam.sanger.ac.uk/)

band number	ORF	product	mature protein length	family, domain, motif	start-end (residue)	E value	Pfam accession no.
1	asr1134	hypothetical protein	59	CsbD	4–56	2.9×10^−14^	PF05532
	asr4653	hypothetical protein	59	CsbD	5–56	4.2×10^−16^	PF05532

2	asr4319	PsaE	69	PSI_PsaE	1–61	8.9×10^−37^	PF02427

4	asr3935	DNA binding protein HU	94	DNA binding	1–89	2.7×10^−33^	PF00216

5	alr0198	unknown protein	91	unknown	—	—	—

6	alr0600	unknown protein	91	Wbp11 DUF2130	43–85	2.1×10^−3^	PF09429
					11–84	2.7×10^−2^	PF09903

7	alr0896	unknown protein	93	Latarcin	23–71	7.6×10^−2^	PF10279

8	all0459	unknown protein	83	unknown	—	—	—

10	alr1718	unknown protein	121	KGG	7–28	3.5×10^−12^	PF10685
					38–53	3.4×10^−5^	
					54–72	2.1×10^−4^	

12	all0615	unknown protein	182	DUF892	15–170	6.6×10^−43^	PF05974
	all4201	50S ribosomal protein L6 Rpl6	182	Ribosomal L6	10–81	7.7×10^−24^	PF00347
					89–164	3.1×10^−29^	

13	all4377	RNA binding protein RbpG	165	RNA recognition	4–75	7.0×10^−5^	PF00076

15	all1861	unknown protein	205	Bacterial SH3	10–43	1.6×10^−6^	PF08239
				Putative peptidoglycan binding	95–131	2.6×10^−12^	PF01471
					153–202	1.1×10^−12^	
	all4782	unknown protein	123	unknown	—	—	—

16	alr0114	hypothetical protein	243	Tic22	1–221	1.1×10^−41^	PF04278

17	alr0806	unknown protein	133/134	unknown	—	—	—

18	alr3276	Peptidase	262	Peptidase_M23	155–253	1.2×10^−29^	PF01551

21	all3826	unknown protein	210	Putative peptidoglycan binding	13–59	1.1×10^−7^	PF01471
					65–120	4.8×10^−12^	
