# Supplementary material for: Cell Surface-Associated Proteins in the Filamentous Cyanobacterium Anabaena sp. strain PCC 7120
Source: Microbes Environ. 2012 Oct 10;27(4):538–43. doi: 10.1264/jsme2.ME12091 (PMC4103569; doi:10.1264/jsme2.ME12091)
Supplement: Supplementary file 1 [file 27_538_s1.pdf]

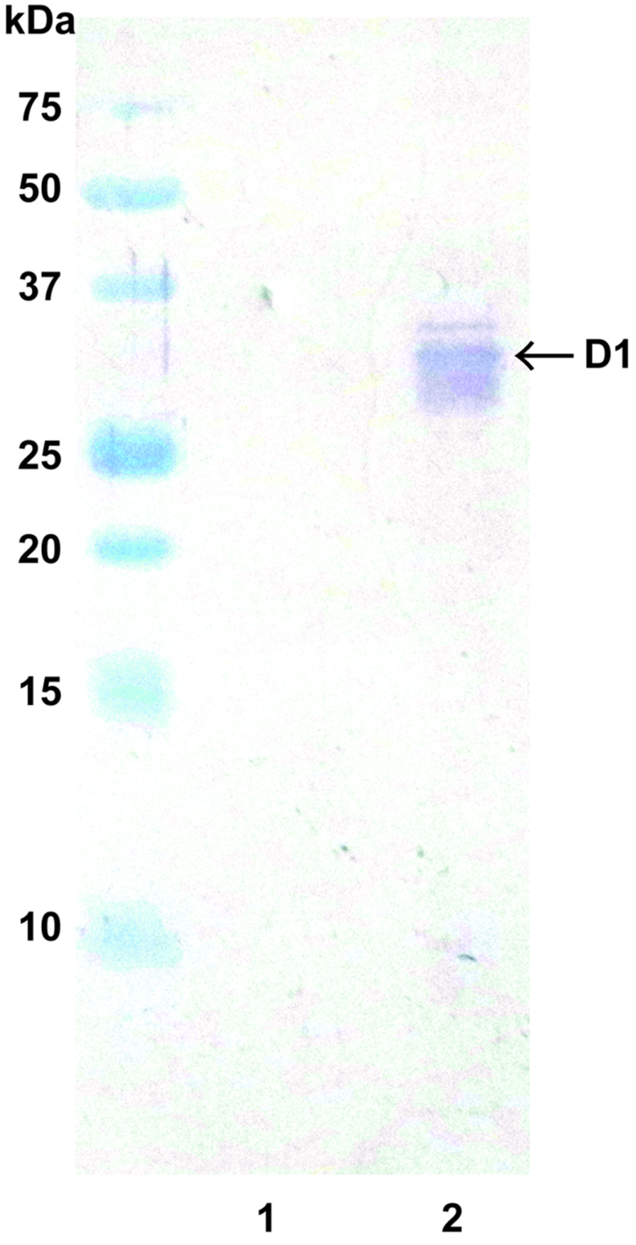

Figure S1 Yoshimura et al.

Western blotting analysis. Lane 1, cell surface-associated proteins; and lane 2, whole cell extract of *Anabaena* sp. strain PCC 7120. The antibody against D1 protein was used (Ikeuchi M and Inoue Y [1987] Specific 125I labeling of D1 [herbicide-binding protein]: An indication that D1 functions on both the donor and acceptor sides of photosystem II. FEBS Lett. 210:71-76). The extraction of cell surface-associated proteins is described in the text. Whole cell extract was prepared by disruption of cells with glass beads in 50 mM Tris-HCl (pH 8.0), 100 mM NaCl, 1 mM EDTA. Protein concentrations were determined by the Brad ford method. Ten μg of each sample was loaded onto a 15% SDS-polyacrylamide gel. After SDS-PAGE, proteins were transferred to a PVDF membrane (GE Healthcare Bioscience, Tokyo, Japan). The membrane were blocked by incubation with 1% (w/v) BSA in 50 mM Tris-buffered saline, pH 8.0, containing 0.05% (v/v) Tween 20 (TBST) for 1 h, and reacted with a 1:5,000 dilution of the rabbit antibody against the D1 protein with shaking for 1 h in 1% (w/v) BSA/TBST. After washing with TBST, the membrane was reacted with a 1:5,000 dilution of alkaline phosphatase-conjugated anti-rabbit immunoglobulin G for 1 h in 1% (w/v) BSA/TBST, followed by washing and subsequent color development with NBT-BCIP (Thermo Fisher Scientific Inc., USA). Color development was carried out for longer (30 min) than the normal discrimination time to check for contamination by cell lysis.
